# Radiolabelling of Extracellular Vesicles for PET and SPECT imaging

**DOI:** 10.7150/ntno.51676

**Published:** 2021-02-13

**Authors:** Azalea A. Khan, Rafael T. M. de Rosales

**Affiliations:** Dept. of Imaging Chemistry and Biology, School of Biomedical Engineering and Imaging Sciences, King's College London, St. Thomas' Hospital, London, United Kingdom.

**Keywords:** Exosomes, EVs, Nanomedicine, Imaging, Radiolabelling, PET, SPECT.

## Abstract

Extracellular vesicles (EVs) such as exosomes and microvesicles have gained recent attention as potential biomarkers of disease as well as nanomedicinal tools, but their behaviour *in vivo* remains mostly unexplored. In order to gain knowledge of their *in vivo* biodistribution it is important to develop imaging tools that allow us to track EVs over time and at the whole-body level. Radionuclide-based imaging (PET and SPECT) have properties that allow us to do so efficiently, mostly due to their high sensitivity, imaging signal tissue penetration, and accurate quantification. Furthermore, they can be easily translated from animals to humans. In this review, we summarise and discuss the different studies that have used PET or SPECT to study the behaviour of EVs *in vivo*. With a focus on the different radiolabelling methods used, we also discuss the advantages and disadvantages of each one, and the challenges of imaging EVs due to their variable stability and heterogeneity.

## Introduction

Extracellular vesicles (EVs) are cell-derived phospholipid bilayer enclosing vesicles. Once thought to be 'garbage bags' used by cells to excrete unwanted molecules, our current understanding of their function includes cell-to-cell communication and expanded their potential applications to the fields of medical diagnostics and therapy 1-4. One of the earliest reports on the potential of EVs as therapeutics was in 1996, when EVs were shown to trigger an adaptive immune response 5, but it wasn't until 2007 that the first demonstration that EVs carry functional RNA, and can deliver it to other cells was reported 6. Since then EVs have been shown to contain a variety of other cell-derived cytosolic molecules, such as proteins, lipids, nucleic acids, among others 7. Consequently, later research revealed the important role EVs play in cell-cell communication 4, 8 and their association in various diseases, such as cancer and metastasis 9-11, neurodegenerative diseases 12, 13, diabetes 14, and inflammatory conditions 15-17. Recent advances in EV engineering have also demonstrated the possibility of chemically modifying these cell-derived vesicles to improve their therapeutic potential, including the introduction of targeting vectors, stability groups (*e.g.* PEGylation) as well as the possibility of drug loading, expanding their therapeutic potential even further 18. For all these reasons, EVs have gained substantial recent attention as potential biomarkers of disease as well as nanotherapeutics.

## Classification of EVs

**Figure 1:**
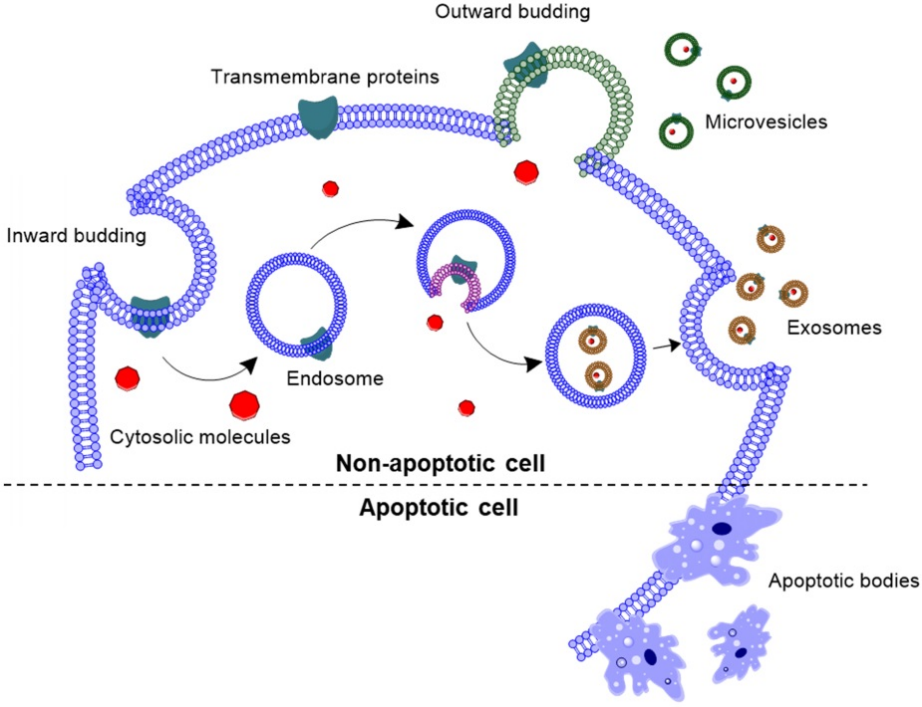
** Biogenesis of extracellular vesicles**. Exosomes are formed by inward budding of the endosomal membrane, followed by being released into the extracellular space. Whereas, microvesicles are formed by outward budding of the cell membrane, and apoptotic bodies are formed by outward blebbing of apoptotic cell membrane.

EVs have been classified in three subtypes, based on their cellular origin as well as their size **(Fig. 1)**: (*i*) exosomes (30-150 nm), (*ii*) microvesicles (50-1000 nm), and (*iii*) apoptotic bodies (500-2000 nm). Exosomes are nanovesicles that are formed by inward budding of the endosomal membrane, and released into the extracellular space by exocytosis 19, 20. Exosomal membrane is enriched with tetraspanins, such as CD63, CD81 and CD9; as well as endosomal proteins, such as Alix and TSG101 7. Unlike exosomes, microvesicles are formed by outward budding of the cell membrane with abundant presence of phosphatidylserine in the outer layer of the bilayer 21, 22. Apoptotic bodies are blebs formed when cells are undergoing apoptosis. However, a recent review outlined that apoptotic bodies are more than just membrane blebs, but are important modulators of immune response 23.

Theoretically, different EVs can be isolated from body fluids or cell culture supernatant by using different (ultra)centrifugation speeds 3, size exclusion chromatography (SEC) or other techniques 24. However, isolation of individual EV populations is challenging due to their overlap in size and complex physicochemical properties. For example, common exosome biomarkers CD63, CD81 and CD9 have been reported to be present in other EV sub-groups dependent on the cell type 25, 26. Hence, adhering to the Minimal Information for Studies of Extracellular Vesicles (MISEV) 2018 guidelines, in this review EVs would be identified by their size 27. Vesicles of sizes < 200 nm would be defined as small EVs (sEVs), vesicles in the 200-500 nm size range would be defined as medium EVs (mEVs), and vesicles > 500 nm would be defined as large EVs (lEVs).

## EVs as nanomedicines

Of the different subtypes of EVs described above, sEVs or exosomes (< 200 nm) have been explored for their potential as therapeutic nanomedicines 28-30. The goal of nanomedicine is to improve the therapeutic effects of drugs by improving targeting to disease sites and/or reducing their systemic toxic side effects 31. Several properties make sEVs an attractive platform for nanomedicine. Compared to other platforms of synthetic origin (*e.g.* liposomes, polymers), natural EVs are antigen presenting, and depending on their cell of origin may have specific tissue-targeting properties 5, 32, 33. sEVs can also be engineered to express ligands for better tissue/cell targeting and stability 34, 35, can be used as drug delivery vehicles 36, 37, and have been reported to accumulate in tumours due to the enhanced permeability and retention (EPR) effect 38, 39. Another important property often cited is their innate ability of crossing the blood-brain barrier, which is a significant biological barrier for other nanomedicinal drug delivery platforms 40-42. sEVs from immune cells may have therapeutic properties by themselves. For example, neutrophil-derived sEVs have been shown to induce inflammatory resolution 43, while sEVs containing foreign antigens/molecules have the ability to trick the immune system, as is the case for many viral diseases 44. Recent reports have also shown that sEVs can be more efficient as gene therapy and drug delivery vehicles than systems based on liposomes 45-47. All these exciting results have resulted in many EV-based therapies progressing quickly towards clinical trials, with over 15 trials registered on NIH ClinicalTrials.gov, including 3 studies looking into treatment for COVID-19, and successful completion of six studies 48-53. However, there is still a lack of information regarding their fate *in vivo* after administration, particularly when these investigational therapeutic nanomedicines are used in humans.

To tackle this lack of information and efficiently develop EV-based therapies, it is important to integrate imaging-based *in vivo* tracking techniques early in the developmental process, to help answer questions regarding their fate after administration into patients/subjects. This will facilitate not only their development but also the progress of clinical trials, allowing the early identification of the most suitable candidates to take forward. As a developmental tool, non-invasive imaging of EV therapies would be an ideal method to monitor and quantify EV biodistribution over time and enable elucidation of their pharmacokinetic profiles. Furthermore, taking into account patient and disease heterogeneity, integrating imaging with EV-based therapies could provide a tool to identify patients/lesions that are likely to respond to the treatment. Such personalised medicine approach would allow optimisation of treatment strategies to suit individual patients' needs 54.

## Imaging EVs *in vivo*

Of the several imaging techniques that have been utilised to track EV biodistribution over time, optical imaging (OI) is the most popular for its simplicity, and for being both cost and time effective. For these reasons, it has been used in many studies to understand and characterise EV properties 55. Nonetheless, OI suffers from inherent physical limitations, mainly poor tissue depth penetration hence its application is limited to the preclinical setting (for whole-body animal imaging), clinical intraoperative and *in vitro*/*ex vivo* imaging. Other imaging techniques don't suffer from this limitation, particularly computed tomography (CT), magnetic resonance imaging (MRI), and the nuclear medicine techniques - single photon emission computed tomography (SPECT) and positron emission tomography (PET). All of these allow whole-body imaging with unlimited imaging signal depth penetration 56. Furthermore, they are available in both the preclinical and clinical setting.

**Figure 2:**
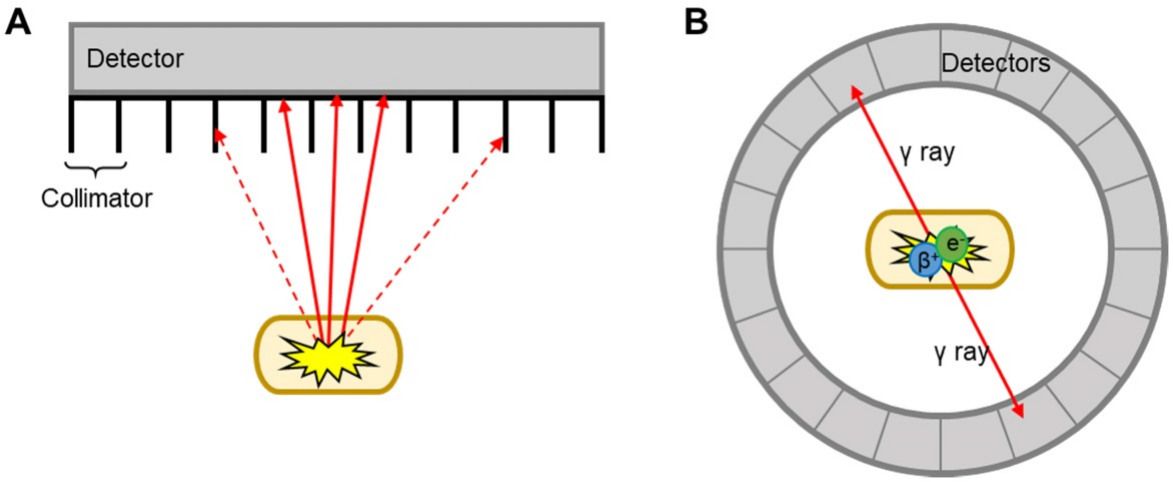
** Schematic representation of SPECT and PET detection. A)** Linear SPECT detector with collimators; dotted arrows = photons that are absorbed by the collimators, solid arrows = photons that reach the detector. **B)** Circular ring of PET detectors detect photons arriving simultaneously in opposite directions.

MRI and CT benefit from excellent spatiotemporal resolution but suffer from low sensitivity (*i.e.* large amount of contrast agent required to allow detection). This is a major limitation for EV imaging, as the concentration of contrast agent per EV required for efficient imaging signal can be potentially damaging to the vesicles. This is perhaps one of the reasons behind the low number of studies that used MRI for EV imaging 57-62. CT has only been used in combination with nuclear imaging techniques (SPECT or PET) for EV imaging. These two techniques offer higher sensitivity compared to CT/MRI (*ca.* 10^6^-fold), no background signal, and allow whole body imaging with accurate signal quantification. By radiolabelling EVs with appropriate radioactive isotopes, or radionuclides, it is possible to track/image EVs using either SPECT or PET. The main differences between SPECT and PET imaging lies in the type of radioisotope used, and how the signal is detected and converted into 3D images (**Fig. 2**). SPECT detect radionuclides that decay by emission of γ photons. These γ photons have definite energies; for example ^99m^Tc, the most used radionuclide in nuclear medicine with a decay half-life (*t*_1/2_) of 6 h, emits 140 keV γ photons. These radionuclides are detected by a rotating gamma camera to generate a 3D image. Collimators are used to only allow radiation at certain angles to reach the detector and thus determine the source **(Fig. 2A)** 63. PET, on the other hand, uses radionuclides that decay by positron (β^+^) emission. Positrons travel for a short distance (depending on their energy, sub-mm to several mm) before annihilating with an electron, an event that produces two γ rays of equal energy (511 keV) simultaneously in opposite directions. These γ photons are detected by a circular ring of detectors that maps out simultaneously arriving γ rays at 180°, a technique known as coincidence detection **(Fig. 2B)** 64. These differences in detection techniques and instrumentation lead to differences in image quality and sensitivity. In general terms, clinical PET is superior to SPECT in some aspects as outlined in **Table 1** below. SPECT, however, has the major advantage that allows multi-radionuclide imaging with radionuclides that emit γ rays of different energies.

**Figure 3:**
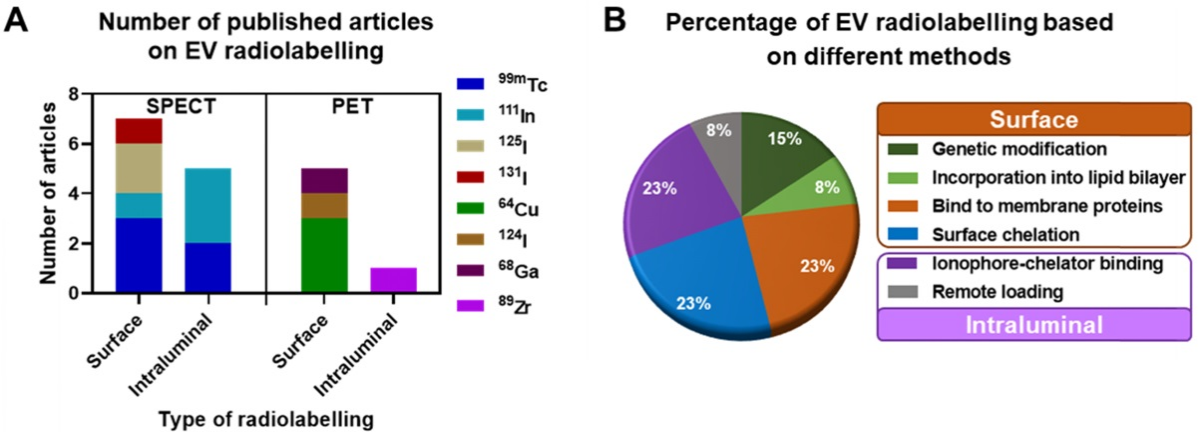
**Research articles published until July 2020. A)** EV radiolabelling with various SPECT and PET radionuclides employing either surface or intraluminal radiolabelling strategy. **B)** Percentage of publications using different techniques to achieve surface and intraluminal radiolabelling. Please refer to the supplementary information for methodology.

In this review we focus on the different methods explored to date to radiolabel EVs that allow *in vivo* tracking with both PET and SPECT imaging. We critically review the main advantages and disadvantages of each method, and where possible discuss them into the context of their *in vivo* imaging properties. For reviews on imaging EVs using other imaging techniques, we point the reader to excellent publications available 41, 55, 66, 67. A total of 16 published articles and pre-prints that included radiolabelling of EVs and EV-mimetics were found (July 2020, see Supporting Information for methodology) and analysed in this review, of which 11 were on SPECT imaging and 5 of them on PET imaging. These articles were further classified into the different types of radiolabelling methods discussed below **(Fig. 3)**.

## Radiolabelling of EVs

**Figure 4:**
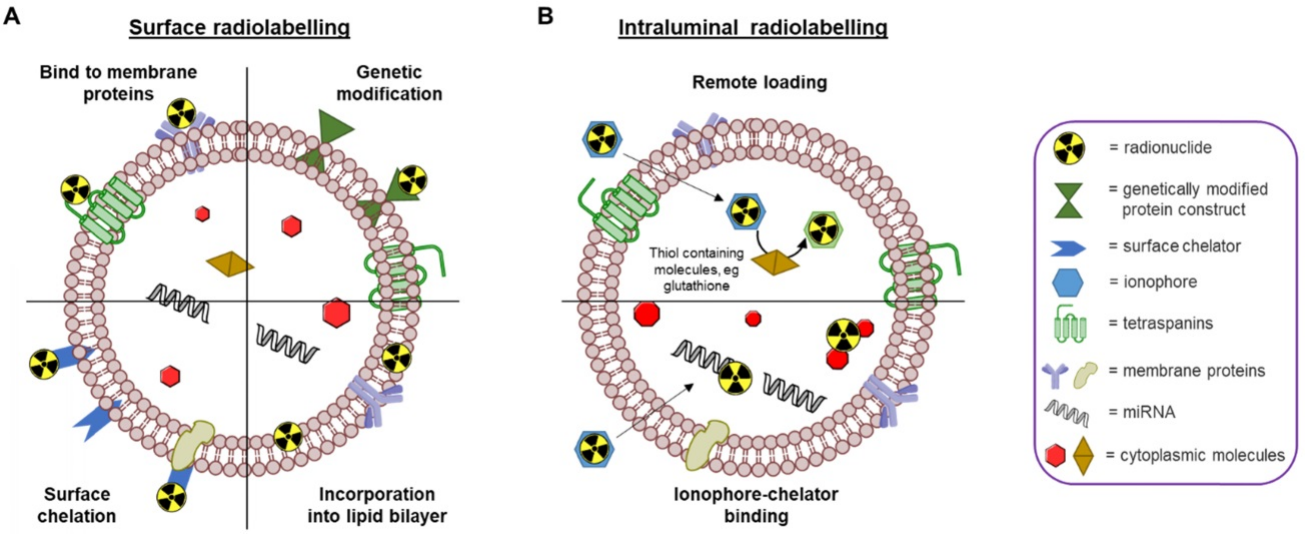
** Schematic representation of different EV radiolabelling methods. A)** *Surface radiolabelling*: radionuclide can be incorporated into the EV membrane directly or *via* a chelator. **B)** *Intraluminal radiolabelling*: ionophores allow radionuclides to be transported across the lipid membrane where they can be trapped as their lipophilicity changes or bind to metal chelating biomolecules.

Due to the similarities in their physical structures, the different chemical concepts that allow radiolabelling of liposomes can also be applied to EVs 68. Specifically, both EVs and liposomes are similar in size and consist of a phospholipid bilayer enclosing an aqueous core capable of carrying a chemical cargo. A recent review by Man *et al.* outlines the different methods that have been employed to radiolabel and image liposomal systems to date, including their pros and cons 69. Similar to liposomes, there are also two primary methods for radiolabelling EVs - surface and intraluminal - that we will briefly describe below **(Fig. 4)**.

### Surface radiolabelling

Surface or membrane radiolabelling is the most common method to radiolabel EVs. This involves incorporation of the radionuclide directly into the lipid membrane or attached to the membrane proteins, either directly or *via* covalent chemical bond formation. Four main methods have been applied to achieve this: 1) genetic modification, 2) direct incorporation of radionuclide into the membrane, 3) radionuclide attachment *via* a chelator (*i.e.* radiometal-binding chemical group) on the surface, and 4) direct incorporation of radionuclide into membrane proteins **(Fig. 4A)**. Direct incorporation of radionuclides relies on non-specific affinities between the radionuclides and the EV membrane components. For the surface chelation methods the radiotracer is conjugated to the membrane, typically, via surface amine groups using standard bioconjugate chemistry techniques. More detailed descriptions behind these methods are described in the 'Radiolabelling of EVs using SPECT radionuclides' and 'Radiolabelling of EVs using SPECT radionuclides' sections below with the corresponding radiotracers (*vide infra*). A potential limitation of surface radiolabelling methods in general is that they could compromise the integrity of the EV's surface, particularly if they involve chemical modification of membrane proteins, or substantial modification of the membrane composition. The importance of EV surface proteins and lipids in their behaviour has been demonstrated in various studies 32, 70-74, and a recently published review described the different methods to modify the surface of EVs 75. These alterations are usually applied to understand EV function or to improve tissue targeting; hence those aimed at enabling radiolabelling have significant potential to alter their natural physicochemical properties and biodistribution.

### Intraluminal radiolabelling

An alternative approach to radiolabel EVs is to entrap the radiotracer inside the intra-vesicular space **(Fig. 4B)**. Thus, the lipid bilayer membrane is expected to protect the radionuclide from trans-chelation by extra-vesicular components, such as serum proteins. This is in contrast to surface radiolabelling, where the radionuclide is more exposed to extra-EV trans-chelation. To radiolabel EVs intraluminally, the radionuclide needs to cross the lipid bilayer and stay within the EV. To achieve this, two methods have been explored to date: 1) remote loading, and 2) ionophore-chelator binding. The first method takes advantage of endogenous intravesicular glutathione that is capable of transforming some complexes, such as [^99m^Tc]Tc-hexamethylpropyleneamine oxime ([^99m^Tc]Tc-HMPAO), from lipophilic to hydrophilic 76. Once the lipophilic radiotracer complex passes through the lipid bilayer membrane, it is converted into its hydrophilic form and thus, gets trapped inside the aqueous core of the EV. The ionophore-chelator binding method exploits well-known ionophore ligands, such as tropolone and 8-hydroxyquinolin (oxine), that form a metastable and neutral complex with the radiometals, allowing them to be transported across the lipid membrane. This method is commonly used to radiolabel cells as well as liposomal nanomedicines, where the radiometal can bind to metal chelating moieties within the liposomal cargo 77. In the case of EVs, the radiometal is expected to bind to intravesicular proteins and/or nucleic acids. The main disadvantage of intraluminal radiolabelling approaches, especially those based on ionophores, is that we lack the knowledge of exactly which component of the EVs' intraluminal space the radionuclide binds to. This may complicate interpretation of *in vivo* images, particularly at late timepoints when EV lipid bilayer fragmentation may be significant and due to the fact that some radionuclides - particularly radiometals such as ^64^Cu - that accumulate in the same organs as EVs (*e.g*. liver, spleen) 69.

The two main radiolabelling categories described above, surface and intraluminal, have been employed with both SPECT and PET radionuclides with various degrees of radiolabelling capabilities. In the next sections we will describe the different EV radiolabelling studies reported to date, that we have classified in to SPECT radionuclides **(Table 2)** and PET radionuclides **(Table 3)**.

## Radiolabelling of EVs using SPECT radionuclides

**Figure 5:**
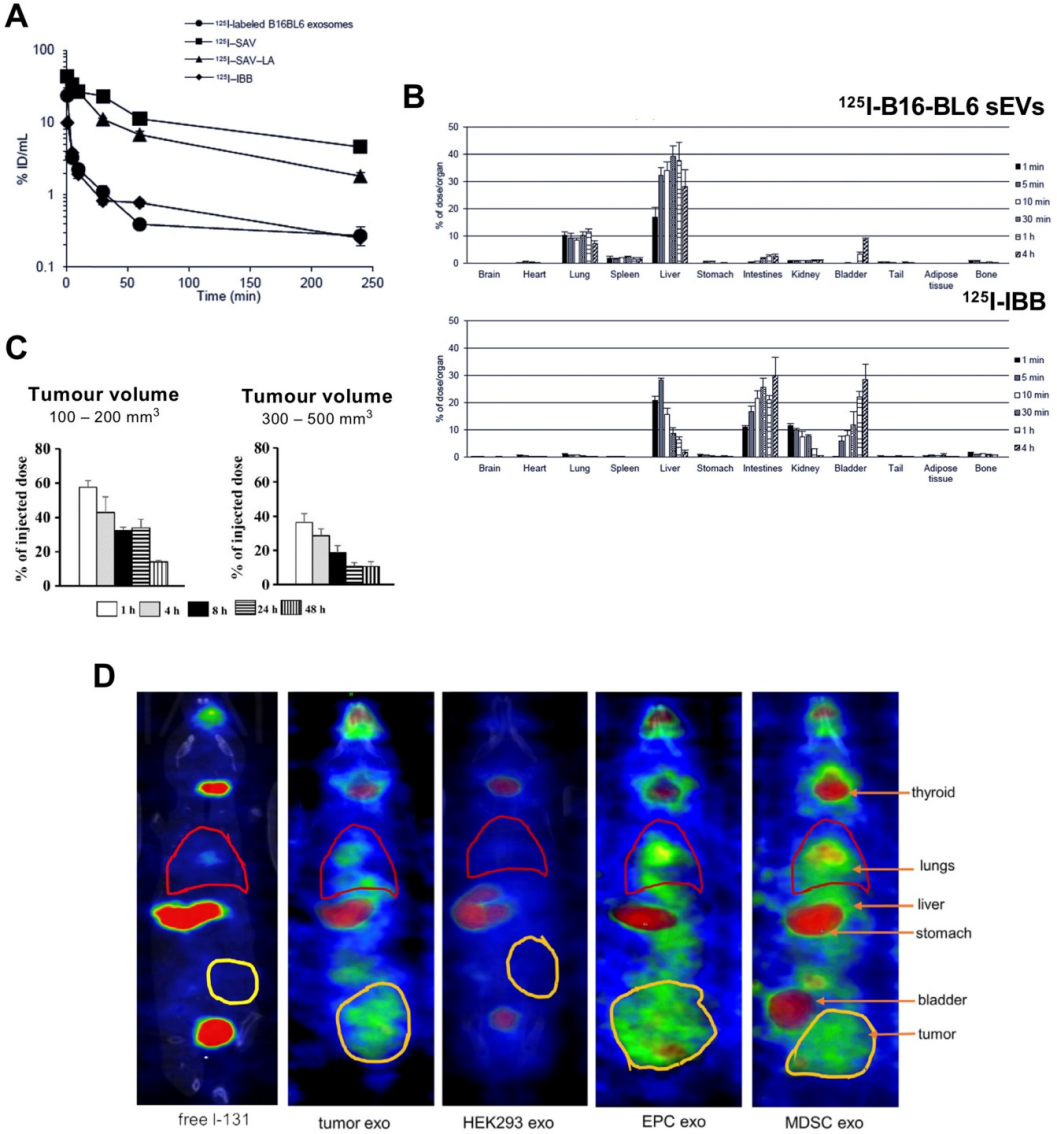
** (A)** Blood clearance profile of ^125^I-labelled B16-BL6 sEVs, [^125^I]I-SAV (streptavidin construct), [^125^I]I-SAV-LA (streptavidin-lactadherin fusion protein), and [^125^I]I-IBB (biotin conjugated radiotracer) in healthy mice after iv. injection; data presented as mean ± standard error of means (SEM) of n = 4. **(B)** *Ex vivo* biodistribution of ^125^I-labelled B16-BL6 sEVs and [^125^I]I-IBB over 4 h post iv. injection; data presented as mean ± SEM of n = 4. Figure taken with permission from Morishita *et al.* 78 **(C)** Retention of intratumorally injected ^125^I-labelled B16-BL6 sEVs in tumor tissues of a xenograft mouse model with tumour volume of 100-200 or 300-500 mm^3^; data presented as mean ± SEM of n = 4. Figure adapted with permission from Matsumoto *et al.* 79 **(D)** *In vivo* biodistribution of ^131^I-labelled EVs (exo) isolated from 4T1 (mouse breast tumour) cells, HEK-293 (human embryonic kidney-293) cells, endothelial progenitor cells (EPC) and myeloid derived suppressor cells (MDSC) compared to free ^131^I biodistribution in tumour bearing mice. Figure adapted with permission from Rashid *et al.* 80.

The first report reporting radiolabelling of sEVs was by Morishita *et al.* in 2014 (publication in 2015, but available online from Nov 2014) 78. Using plasmid transfection, lactadherin - a glycoprotein found on cell membrane - was replaced with streptavidin on B16-BL6 cells and cultured for sEV isolation. Streptavidin containing B16-BL6 sEVs (70 ± 3 nm) were radiolabelled with biotin-conjugated ^125^I (*t_1/2_* = 59.4 d), [^125^I]I-IBB. Strong binding affinity (K_d_ ~10^15^ M^-1^) makes the streptavidin-biotin conjugate a popular pretargeting method in radioimmunotherapy 94. The radiolabelling yield (RLY) of B16-BL6 sEVs with [^125^I]I-IBB was ~80%, and *in vitro* serum stability was ~95% for up to 4 h. Despite blood clearance of ^125^I-labelled B16-BL6 sEVs having the same profile as the free radiotracer ([^125^I]I-IBB) in healthy mice **(Fig. 5A)**, their *ex vivo* biodistributions were significantly different with ^125^I-labelled B16-BL6 sEVs showing accumulation in liver, lung and spleen **(Fig. 5B)**. Surprisingly, ^125^I uptake in the thyroid was not mentioned in the paper. Thyroid is an organ of interest when using iodine as a sign of radiochemical instability with iodinated radiotracers, due to the presence of the human Na^+^/I^-^ symporter (hNIS). Stomach uptake, another organ with high affinity for free iodide *via* hNIS was very low, suggesting *in vivo* radiochemical stability during the 4 h study. This study, however, is the only study to carry out mathematical pharmacokinetic modelling of radiolabelled sEVs. The same group later published another study based on the same radiolabelling method 79, and reported higher retention of sEVs in xenograft tumours of up to 200 mm^3^ volume compared to larger tumours of up to 500 mm^3^ when injected intratumorally **(Fig. 5C)**.

Although useful as a preclinical tool, genetic modification is a challenge in terms of clinical translation. Radiolabelling with iodine can easily be performed using iodination beads (iodo-bead method), which consists of a polystyrene bead coated with an oxidising agent facilitating the reduction of tyrosine residues and iodine substitution 95, thus iodinating EV surface proteins. Using this method, Rashid *et al.* achieved > 80% RLY with ^131^I (*t_1/2_*= 8 d) for sEVs derived from 4T1 cells 80. Although up to 80% of the radiolabelled sEVs were stable in serum for 24 h; *in vivo* imaging at early time-points (*ca.* 3 h) showed high thyroid, stomach and bladder uptake for both tumour cell- and healthy cell-derived sEVs, which correlates with release of free ^131^I **(Fig. 5D)**. It has been previously reported that radio-iodination using the iodo-bead method is prone to rapid deiodination *in vivo* as early as 2 h post injection 96. Hence, it seems that sEVs radiolabelled using the iodo-bead method suffer from low *in vivo* radiochemical stability and cannot reliably be used to determine their biodistribution.

**Figure 6:**
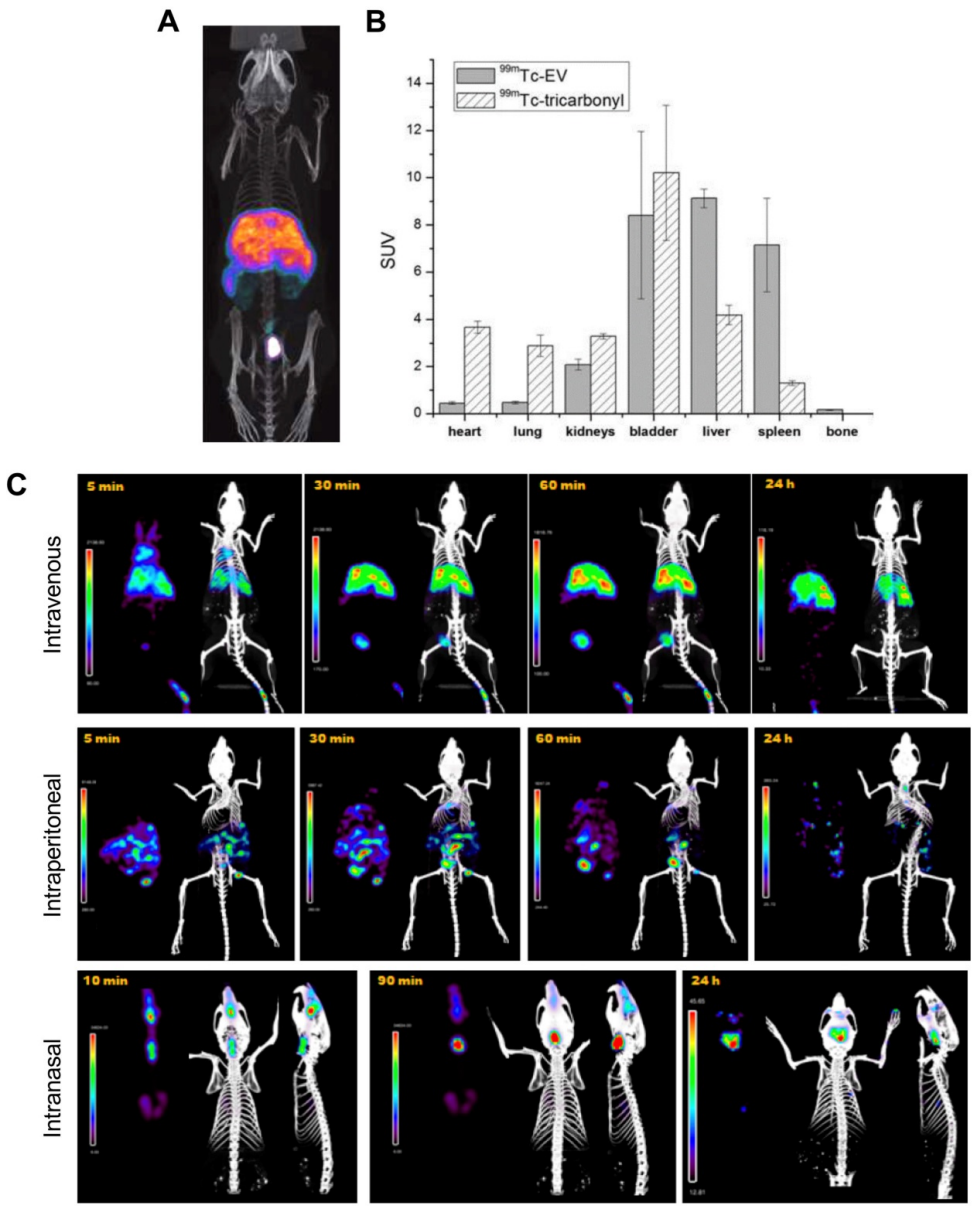
** A)** SPECT-CT images of [^99m^Tc]Tc-tricarbonyl labelled RBC derived sEVs. **B)** *Ex vivo* biodistribution of ^99m^Tc-sEVs and [^99m^Tc]Tc-tricarbonyl injected iv. in male BALB/c mice about 2 h post injection (n = 3). Figures taken with permission from Varga *et al.* 81 **C)** *In vivo* biodistribution of ^99m^Tc-labelled goat milk derived sEVs injected in healthy female BALB/c mice via three different administration routes over time as indicated on the images. Figure adapted with permission from González *et al.* 83.

The long half-lives of radioiodines are ideal for long term *in vivo* tracking of EVs. Nevertheless, ^99m^Tc, with a shorter half-life of 6 h, is the most commonly used radionuclide for imaging of EVs probably due the availability/low cost and favourable radiation properties of this radionuclide for imaging. The first use of^ 99m^Tc-labelled EVs was reported by Varga *et al.*, who used [^99m^Tc]Tc-tricarbonyl ([^99m^Tc(CO)_3_]^+^) to label red blood cell- (RBC) derived sEVs (188 ± 11 nm) 81. Unlike liposomes, that lack appropriate donor ligands and require surface modification 97, EVs should be able to bind to [^99m^Tc(CO)_3_]^+^ *via* surface proteins, most likely involving histidine donors 98. A RLY of 38.8 ± 6.2% was achieved after 30 min incubation at room temperature, a relatively low RLY which is not surprising as efficient binding to this inert complex requires high temperatures incompatible with biomolecules 99. An *in vivo* imaging comparison between [^99m^Tc(CO)_3_]^+^ and [^99m^Tc(CO)_3_]^+^-RBC-sEVs is consistent with efficient radiolabelling and high stability for the latter. However, the short imaging timeframe of the study (< 2 h) does not allow evaluation of the long-term *in vivo* stability of [^99m^Tc(CO)_3_]^+^-RBC-sEVs **(Fig. 6A-B)**. A more recent report also exploited the use of [^99m^Tc(CO)_3_]^+^ to radiolabel human epidermal growth factor receptor 2 (HER2) targeted- HEK 293T sEVs 82. Contrary to the previous study, a high RLY was achieved by incubation of [^99m^Tc(CO)_3_]^+^ with the sEVs at a relatively low temperature of 37 °C for 60 min. *In vivo* imaging showed expected high liver uptake, but no spleen, as well as significant kidney and intestine signal that could potentially indicate free [^99m^Tc(CO)_3_]^+^.

An alternative surface radiolabelling method with ^99m^Tc involves reduction of the unreactive [^99m^TcO_4_]^-^ in the +7 oxidation state to ^99m^Tc^4+^ using stannous chloride (SnCl_2_), a commonly used RBC radiolabelling method 100. Gonzalez *et al.* optimised this method for sEV radiolabelling and achieved 37 ± 9% RLY using 2 mM SnCl_2_ and 75 µg of milk-derived sEVs (122 ± 1 nm) 83. Using this method,^ 99m^Tc was incorporated directly into the milk-derived sEV membrane, although the exact binding site of the ^99m^Tc^4+^ ion is unclear and is likely to be non-specific binding to surface proteins. *In vitro* stability within 48 h in PBS was high, although it would have been interesting to test with more challenging conditions (*e.g.* in the presence of serum components). Looking into different administration routes the authors noted significant differences in the biodistribution depending on the injection route, with mainly liver and spleen uptake *via* intravenous (iv.) administration, unspecific abdominal distribution *via* intraperitoneal administration, and mainly digestive distribution after intranasal administration, with some minor brain uptake **(Fig. 6C)**.

**Figure 7:**
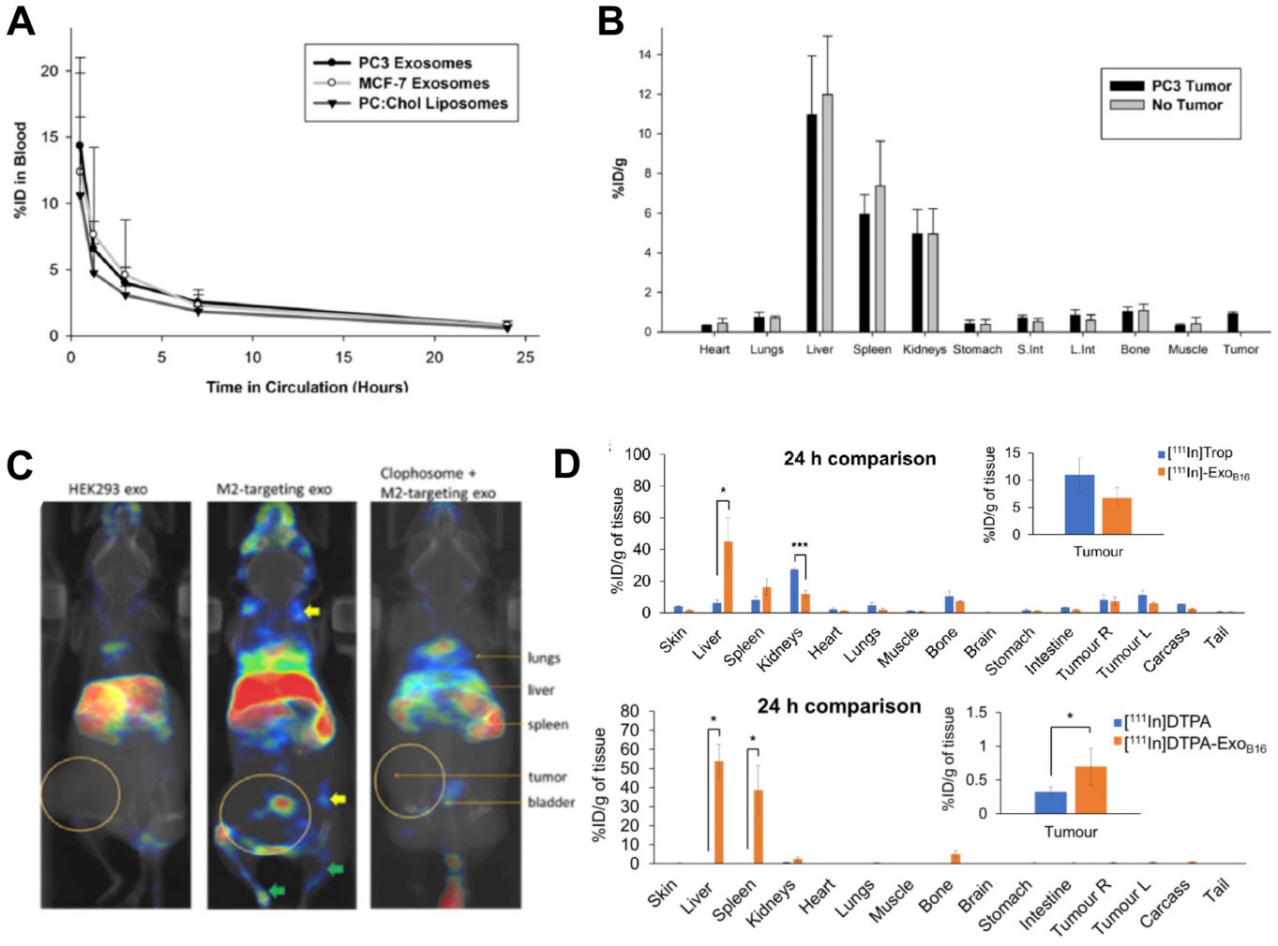
**(A)** Blood clearance of ^111^In-labelled PC3 (prostate cancer) and MCF-7 (breast cancer) sEVs, compared to liposomes**. (B)** *Ex vivo* biodistribution of ^111^In-labelled PC3 sEVs in PC3 tumor-bearing nude mice and non-tumor bearing mice 24 h post injection. Figures taken with permission from Smyth *et al.* 85** (C)** *In vivo* biodistribution of iv. administered [^111^In]In-oxinate labelled CD206-positive M2 macrophage targeting sEVs (middle image) as well as control groups (left and right images) in tumour bearing BALB/c mice at 3 h post injection. Figure taken with permission from Rashid *et al.* 86** (D)** *Ex vivo* biodistribution of iv. administered [^111^In]In-tropolone and [^111^In]In-DTPA labelled B16-F10 sEVs at 24 h post injection. Data presented as mean ± SD of n = 3 and analysed by Student's t-test. Figure adapted with permission from Faruqu *et al.* 84.

Despite being the most widely used radionuclide, the short half-life of ^99m^Tc only allows imaging for up to 24 h post administration, which is not suitable for long term *in vivo* tracking of EVs. This can be resolved by using ^111^In (*t_1/2_* = 2.8 d), another clinically available gamma-emitting radionuclide that is the second most commonly used for EV imaging after ^99m^Tc (**Fig. 3**). Smyth *et al.* used [^111^In]In-oxinate (intraluminal labelling) to radiolabel sEVs derived from PC3 (140 ± 59 nm) and MCF7 (130 ± 57 nm) cells, with RLY of 81% and 67%, respectively 85. These differences in RLY could be due to different affinities of the intraluminal composition of the two types of sEVs for the same radiometal. Blood clearance of the two sEVs was fast, and similar in tumour bearing mice as well as that of liposomes **(Fig. 7A)**. High uptake in liver, spleen and kidneys, but low tumour uptake was reported in *ex vivo* data at 24 h for both healthy and tumour bearing mice **(Fig. 7B)**. High kidney uptake could be a sign of “unchelated” ^111^In release, but this was not evaluated. In a very interesting study, Rashid *et al.* also used intraluminal radiolabelling ([^111^In]In-oxinate) to track CD206-positive M2 macrophage-specific sEVs (106 ± 14 nm) *in vivo* using SPECT imaging 86. The authors used [^111^In]In-oxinate to achieve very high RLY (98%) and *in vitro* stabilities, as assessed by thin layer chromatography. *In vivo* SPECT-CT images using M2-targeted sEVs showed the expected liver and spleen uptake seen with other sEVs, but also interesting uptake in lymph nodes **(Fig. 7C)** - a finding that has also been observed with ^89^Zr-labelled PANC1 sEVs (*vide infra*) 93 - and lungs. In addition, M2-targeted sEVs showed increased uptake in tumours, as well as significant kidney and bladder signal that the authors assign to excretion of radiolabelled sEVs. Although it would have been preferable to report image scales to allow image comparisons and quantify organ/tumour uptake using normalised standard units (*ca*. %ID/mass, %ID/volume, SUV), instead of %ID or counts/mass, these results show the potential of engineering sEVs for cell-specific targeting.

Similar to [^111^In]In-oxinate, [^111^In]In-tropolone is another lipophilic radiotracer that allows intraluminal radiolabelling, but with higher inertness/stability compared to [^111^In]In-oxinate that makes it more resistant to transchelation, and hence potentially less effective for intraluminal radiolabelling. In fact, [^111^In]In-oxinate often results in higher cell RLYs when compared to [^111^In]In-tropolone 101. Faruqu *et al.* used [^111^In]In-tropolone to achieve a RLY of 4.73 ± 0.39% with B16-F10 sEVs (132 ± 6 nm) 84. In this study SEC using Sepharose^®^ CL-2B resin was used to separate unbound radiotracer, a technique that potentially results in losses of *ca.* 50% of the vesicles. The authors also performed membrane radiolabelling of the same sEVs using [^111^In]In-DTPA (RLY = 19.2 ± 4.5%). Overall, the pharmacokinetics and tissue biodistribution differed slightly depending on whether they were radiolabelled on the membrane or intraluminally **(Fig. 7D)**. The relatively lower radiochemical stability of the [^111^In]In-tropolone labelled sEVs was evident in the *in vivo* biodistribution with lower liver/spleen retention. This study also demonstrated that there are no significant differences in sEV biodistribution between immunocompetent (C57BL/6) and immunocompromised (NOD SCID gamma (NSG)) mice, except minor differences in tumour uptake, that could be explained by the smaller population of tumour associated macrophages in NSG mice.

## Radiolabelling of EVs using PET radionuclides

**Figure 8:**
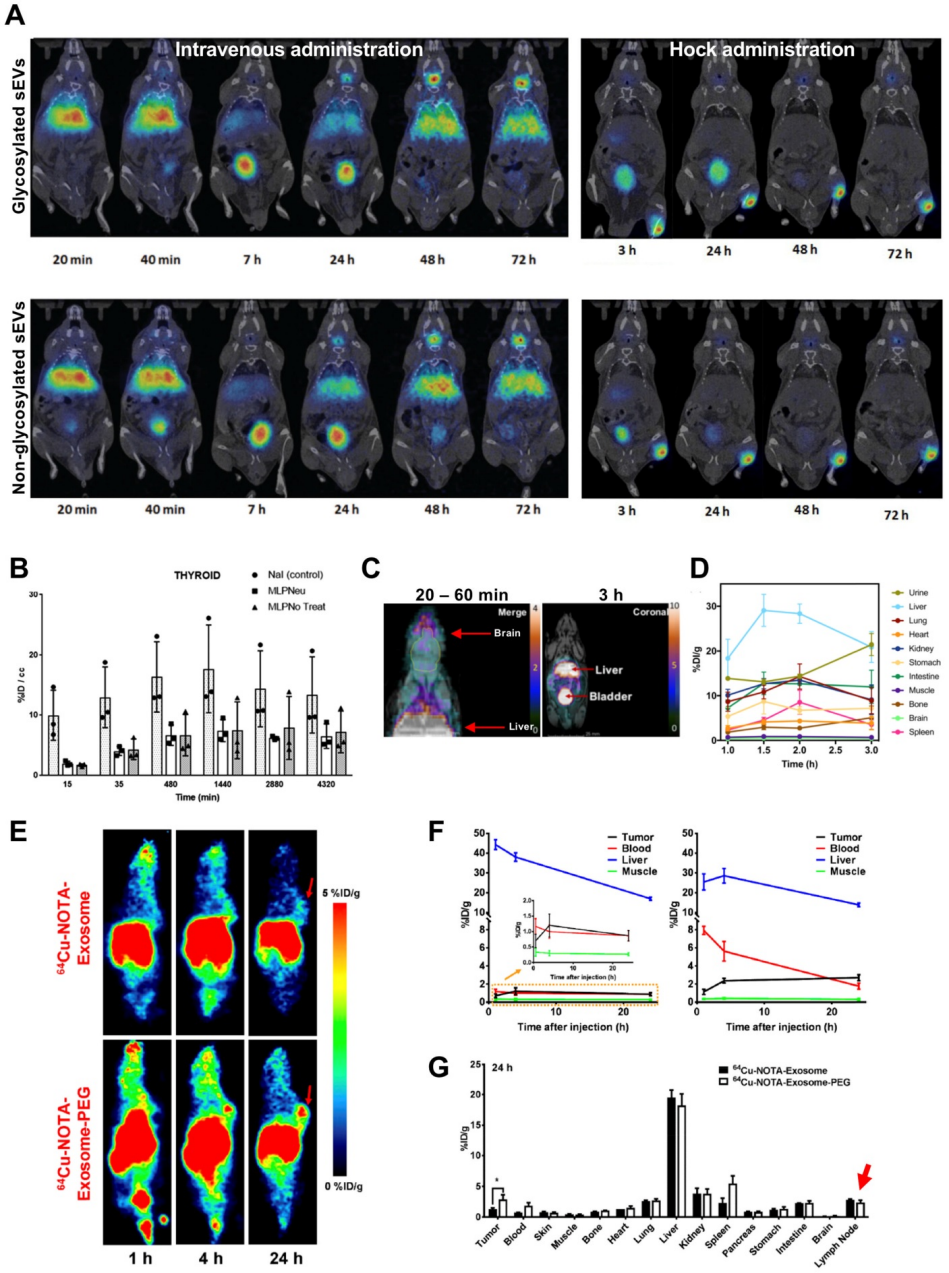
**(A)** *In vivo* biodistribution of ^124^I-labelled MLP29 sEVs following iv. and hock administration. **(B)** *Ex vivo* biodistribution of ^124^I-labelled MLP29 sEVs in the thyroid over time for NaI (control), glycosylated sEVs (MLPNeu) and non-glycosylated sEVs (MLPNo Treat) following iv. administration, data given as mean ± standard deviation (SD) of n = 3. Figures adapted with permission from Royo *et al.* 89 **(C)** PET-MR images of iv. administered [^64^Cu]Cu-DTPA labelled sEVs 20 - 60 min and 3 h post injection. **(D)** *Ex vivo* biodistribution of [^64^Cu]Cu-DTPA labelled sEVs at 1, 1.5 and 2 h post injection; data represented as mean ± SEM of n = 3-4. Figures adapted with permission from Banerjee *et al.* 90 **(E)** *In vivo* PET images, and **(F)** time activity curves of iv. administered [^64^Cu]Cu-NOTA labelled 4T1 sEVs comparing the biodistribution of PEGylated and non-PEGylated sEVs in 4T1 tumour bearing female BALB/c mice over time. **(G)** *Ex vivo* biodistribution of ^64^Cu-4T1 sEVs, PEGylated or non-PEGylated, in 4T1 tumour bearing female BALB/c mice 24 h post injection. Red arrow highlights lymph node uptake. Data expressed as mean ± SD of n = 3. Figures taken with permission from Shi *et al.* 91.

It is well established that intra-vesicular nucleic acids and proteins can be used as biomarkers for various diseases. Recently, it has been reported that the glycan profiles of EVs can also be used as cancer biomarkers 102. Moreover, enrichment of specific glycoproteins, such as sialic acid 103, allow iv. injected EVs to be captured by CD169 positive macrophages in spleen and lymph nodes 104. Royo *et al.* evaluated the effect of glycosylation on sEV biodistribution by radiolabelling them with ^124^I (*t_1/2_ =* 4.2 d) 89. Using iodination tubes (iodogen method), which allows oxidation of ^124^I and thus radiolabels proteins on cell membranes 95, and a RLY of >15%, they have shown that modified glycosylation allows sEVs to accumulate and retain in the lungs after iv. injection; and to migrate through the lymph system after hock injection (injection in the joints), even though a large amount was retained in the injection site **(Fig. 8A)**. The *in vivo* PET imaging data showed that sEVs start accumulating in the liver as early as 30 s after iv. injection, and joint administration results in expected lymphatic drainage. Although it is difficult to compare the *in vivo* images without appropriate scale bars; there were only minor differences (lung uptake) between glycosylated or non-glycosylated sEVs, with both showing increasing signal in bladder and thyroid over time, the latter being a sign of *in vivo* deiodination **(Fig. 8A-B)**. Continuing with membrane labelling, Banerjee *et al.* used [^64^Cu]Cu-DOTA-Maleimide (*t_1/2_ =* 12.7 h) to radiolabel sEVs (~110 nm) 90. DOTA was attached on the sEVs' surface using endogenous membrane thiol groups*.* This allowed *ca.* 20% RLY of umbilical cord blood cell sEVs. Interestingly, *in vivo* imaging showed brain uptake 20 - 60 min post injection **(Fig. 8C)**. A considerable amount of sEV uptake was seen in the bladder, which increased over time as the liver signal decreased **(Fig. 8D)**. Interestingly adding a further SEC purification following ultracentrifugation increased sEV accumulation in liver and spleen but decreased in urine/bladder (discussed in the “Challenges” section below).

Facilitated by the specific glycan profile, the rapid uptake of EVs by liver and spleen, results in very short blood circulation time when injected iv. 105, 106. PEGylation is commonly used in nanomedicine to overcome such issues 107. Likewise, PEGylated EVs have been shown to improve circulation, reduce liver sequestration and improve tumour uptake 108. Shi *et al.* was the first group to study *in vivo* biodistribution of PEGylated EVs, using [^64^Cu]Cu-NOTA 91. Using the same (or less) amount of sEVs (~ 106 nm) as Banerjee and his group, they achieved considerably higher RLY (91.2 ± 0.2% *vs.* ~ 20%). This could be the result of the well-known high radiochemical stability of [^64^Cu]Cu-NOTA complexes 109, 110. PEGylation had the desired effect of increased sEV circulation, decreased liver uptake and increased tumour uptake, particularly after 24 h **(Fig. 8E-F)**. Uptake in the lymph nodes (*ca.* 2% ID/g) for both PEGylated and non-PEGylated was also identified from the *ex vivo* biodistribution data, but not discussed further **(Fig. 8G)**. In another report involving surface radiolabelling with PET radionuclide, Jung *et al.* isolated sEVs from 4T1 breast cancer cells (~ 100 nm) and radiolabelled them with ^64^Cu and ^68^Ga, *via* NOTA-isothiocyanate conjugation to sEV surface amine groups (from surface proteins) and also labelled with Cy7 to allow optical imaging 92. RLY with ^64^Cu was > 98%, which is comparable to what was reported by Shi *et al*. RLY and serum stability of > 95% by TLC were only reported for ^64^Cu-labelled sEVs. Healthy mice were injected iv. or subcutaneously. As expected from this administration route, lymph node uptake was observed after subcutaneous injection, and the PET signal correlates well the fluorescent signal. High uptake in the lungs, liver and spleen was observed for iv. injected sEVs. Furthermore, presence of CD63 and Cy7 positive sEVs in liver, spleen and lymph nodes were confirmed by histological analysis.

**Figure 9:**
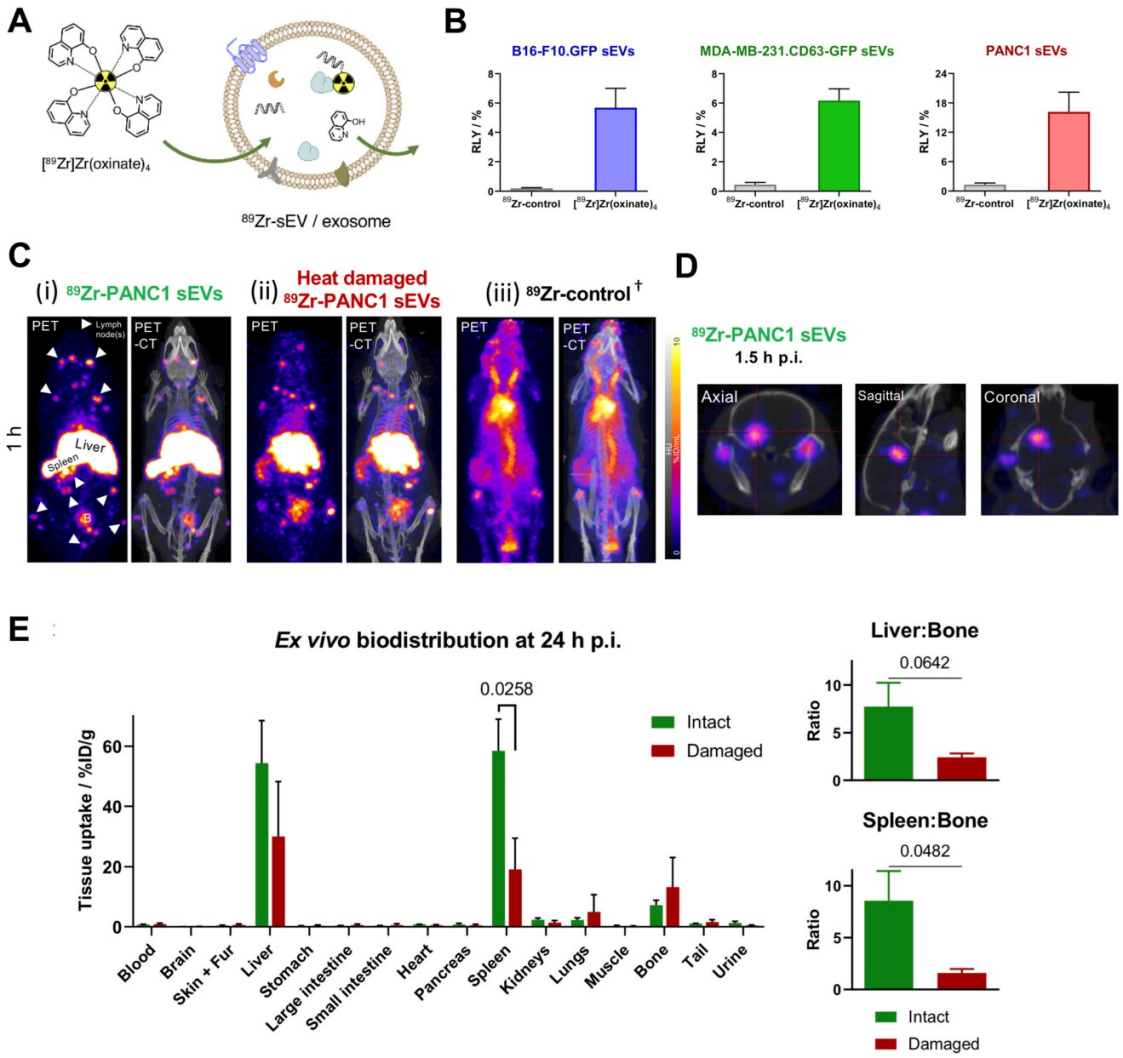
**(A)** Schematic representation of intraluminal radiolabelling of sEVs using [^89^Zr]Zr-oxinate. **(B)** Radiolabelling yield (RLY) of 1x10^10^ B16-F10.GFP sEVs, 1x10^10^ MDA-MB-231.CD63-GFP sEVs and 1x10^11^ PANC1 sEVs; data given as mean ± SD of n = 3. **(C)** *In vivo* PET-CT images of C57BL/6 mice injected iv. with ^89^Zr-PANC1 sEVs, heat-damaged ^89^Zr-PANC1 sEVs and ^89^Zr-control, 1 h post injection; B = bladder, † = PET image scale for ^89^Zr-control is 10 times that of the other images; adjusted for image clarity.** (D)** Image slices of a mouse injected with intact ^89^Zr-PANC1 sEVs showing uptake in brain; image scale is the same as in **C**. **(E)** *Ex vivo* biodistribution of “intact” (n = 3) and “heat-damaged” (n=2) ^89^Zr-PANC1 sEVs. Ratio of liver:bone uptake (n = 3) and spleen:bone uptake (n = 2) is shown on the right; data given as mean ± SD of the n values and analysed by Student's t-test. Figures taken from Khan *et al.* 93.

To date there is one report on intraluminal radiolabelling of sEVs using PET radionuclides 93. Khan *et al.* exploited the proven cell and liposome radiolabelling capabilities of [^89^Zr]Zr-oxinate 77, 111, 112, to radiolabel three different cancer cell-derived sEVs with RLYs as high as *ca.* 20% for 1 x 10^11^ PANC1 sEVs (97 ± 4 nm) **(Fig. 9A-B)**, and *ca.* 76% radiochemical stability in PBS over 26 h. An alternative *in vitro* stability was demonstrated in a cell-uptake study in the presence of serum proteins, showing significant differences between cell lines over 4 h in the uptake of ^89^Zr-PANC1 sEVs *vs.* control groups. *In vivo* PET imaging in healthy mice showed the expected biodistribution of sEVs with uptake in liver and spleen, and interestingly several lymph nodes and brain, particularly at early timepoints (**Fig. 9C-D**). Furthermore, significant differences were observed in the uptake of ^89^Zr-PANC1 sEVs and heat-damaged ^89^Zr-PANC1 sEVs. The latter group was aimed at denaturing the radiolabelled PANC1 sEVs to study the effect in the biodistribution. In particular, significant lower spleen uptake was observed at all time-points, leading to the suggestion that spleen/bone and liver/bone ratios may be used as *in vivo* markers of stability of sEVs radiolabelled using [^89^Zr]Zr-oxinate **(Fig. 9E)**.

## Radiolabelling of exosome mimetic vesicles (EMVs)

Variability in EV production, isolation and radiolabelling properties can be overcome by using exosome mimetic vesicles (EMVs). EMVs are produced mainly by serial extrusion of cell membrane, and so far used for radiolabelling 113, but can also be produced by other methods 68. The main advantage of EMVs is that they can be mass produced in larger concentration than EVs. They are comparable to EVs in size, protein and lipid composition, biomarker expression, and retain tissue targeting properties.

**Figure 10:**
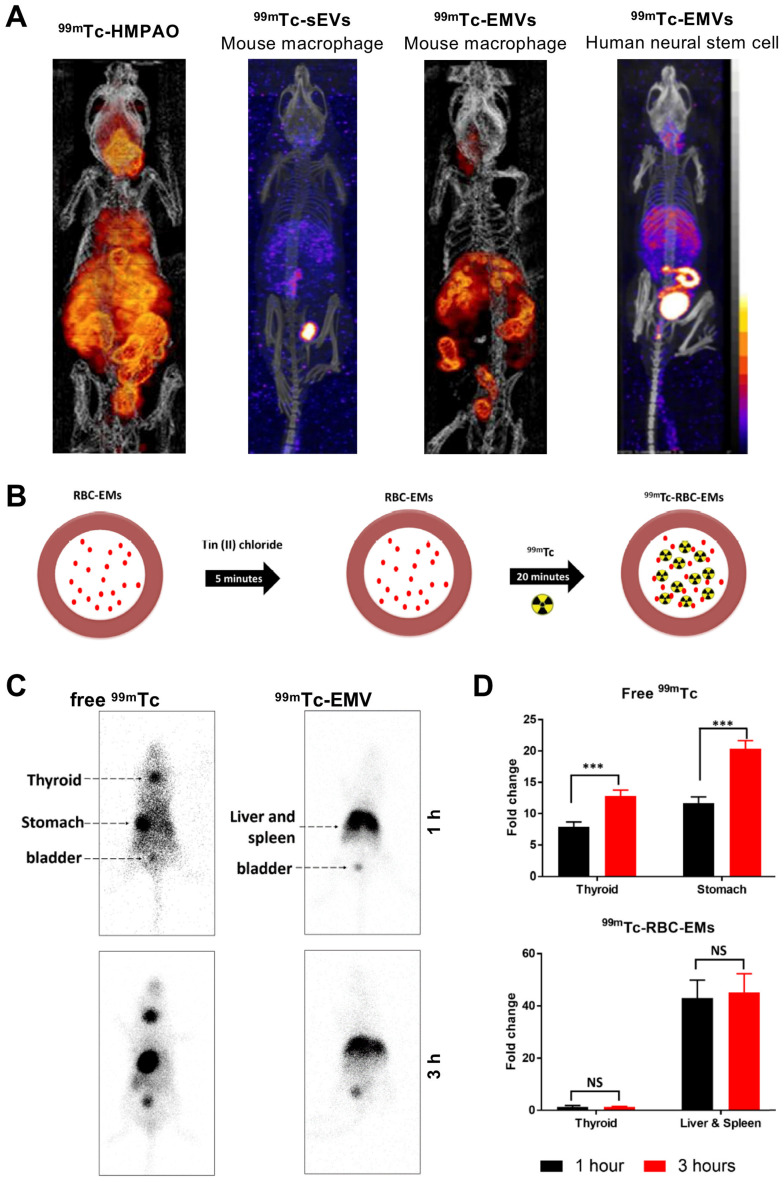
**(A)** *In vivo* SPECT-CT images of ^99m^Tc-labelled EMVs derived from mouse macrophages and human neural stem cells, compared with ^99m^Tc-labelled sEVs derived from mouse macrophages and free [^99m^Tc]Tc-HMPAO 3 h post injection. Figures taken with permission from Hwang *et al.* 87** (B)** Schematic representation of the protocol for radiolabelling red blood cell-derived EMVs with ^99m^Tc*.* **(C)** *In vivo* gamma camera images of iv. administered free ^99m^Tc and ^99m^Tc-EMVs in male C57BL/6 mice 1 h and 3 h post injection. **(D)** Quantification of free ^99m^Tc and ^99m^Tc-EMVs injected in organs of interest 1 h and 3 h post injection; data presented as mean ± SD of n = 4 and analysed by Student's t-test. Figures adapted with permission from Gangadaran *et al.* 88.

Using mouse macrophage derived EMVs (218 ± 8 nm), Hwang *et al.* managed to achieve RLY of >93% with [^99m^Tc]Tc-HMPAO (*t_1/2_ =* 6 h) 87. They utilised the endogenous intra-vesicular thiol groups to convert the lipophilic [^99m^Tc]Tc-HMPAO complex into a hydrophilic one, thus trapping the radionuclide in the intraluminal space, unlike the surface thiol groups used previously by Banerjee *et al*. *In vivo* biodistribution of two different types of ^99m^Tc-labelled EMVs were compared with mouse macrophage derived sEVs, and although comparisons are difficult from the images reported, it seems that significant release of the [^99m^Tc]-HMPAO complex and/or [^99m^TcO_4_]^-^ is evident **(Fig. 10A)**. Later on, Gangadaran *et al.* also studied biodistribution of ^99m^Tc-labelled RBC derived EMVs (201 ± 16 nm). Their radiolabelling hypothesis is based on the RBC radiolabelling protocol, where ^99m^Tc^4+^ reduced by SnCl_2_ binds to intercellular haemoglobin 114. In this case, ^99m^Tc bound to haemoglobin inside the EMVs **(Fig. 10B)**. With ~100% RLY after 20 min incubation, no further purification was used. Healthy C57BL/6 mice were injected iv. with ^99m^Tc-labelled EMVs and gamma camera imaging was performed, as opposed to SPECT. Nonetheless, no significant difference was observed between 1 h and 3 h post injection for the EMVs, unlike for free ^99m^Tc. Once again, significant signal from the liver/spleen and bladder was observed with the radiolabelled EMVs.** (Fig. 10C-D)**. Subsequently, due to the efficiency and stability of radiolabelling, the group used the ^99m^Tc-labelled EMVs to successfully radiolabel and track white blood cells *in vivo* 115.

## Challenges in the radiolabelling and *in vivo* SPECT/PET imaging of EVs

One of the biggest challenges in the *in vivo* imaging of EVs is their instability. A study by Clayton *et al.* demonstrated abundant presence of CD55 and CD59 on EVs, that could lend themselves longer survivability *in vivo* 72. However, according to the EV radiolabelling studies discussed above, as well as studies using non-radiolabelled EVs 104, 116, blood half-life of iv. administered EVs was found to be as short as < 2 min. However, presence of EVs could be detected in the reticuloendothelial system (RES) organs, particularly in liver and spleen, long after clearance from blood.

**Figure 11:**
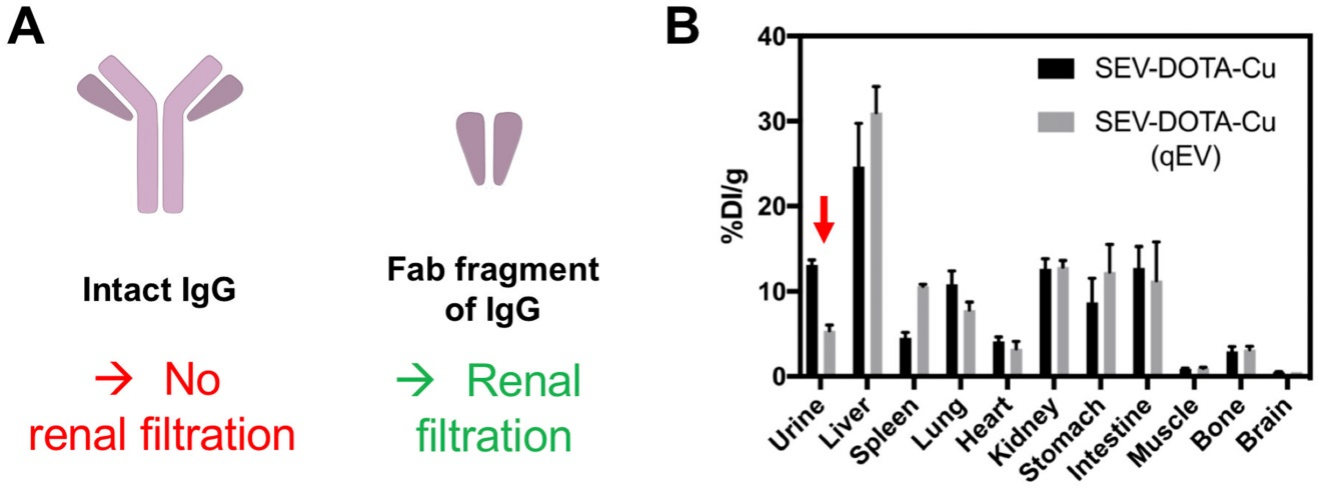
** (A)** Schematic structures of an intact IgG antibody (~150 kDa) and its Fab fragment (~50 kDa). **(B)** *Ex vivo* biodistribution of ^64^Cu-labelled sEVs isolated by ultracentrifugation only *vs.* ultracentrifugation combined with SEC (using a qEV column). Red arrow indicating the difference in urine signal. Figure taken with permission from Banerjee *et al.* 90.

After liver and spleen, the highest accumulation of imaging signals is observed in the bladder; only possible if the EVs are able to pass through the glomerular filtration in the kidneys. Using quantum dots, Choi *et al.* demonstrated that, nanoparticles >8 nm are not typically cleared by the kidneys 117. This study suggested that the renal filtration size threshold maybe comparable to small proteins and was further demonstrated by antibody clearance data. Thus, renal clearance of intact antibodies is considered insignificant because the size of a typical antibody (*e.g.* ~150 kDa for IgG) is much larger than the glomerular filtration threshold (~55 kDa) **(Fig. 11A)** 118. Antibody fragments, on the other hand, are much smaller (for example, the size of the Fab fragment of IgG is ~50 kDa) and are cleared by kidneys 119. Hence it seems to us that the kidney/bladder uptake observed in many SPECT/PET imaging studies with radiolabelled EVs is likely to be the result from fast disintegration of EVs in blood/serum. The radioactive hot-spots seen in bladder in the SPECT and PET images are either from the radiotracer itself (that taking into account that bladder uptake is often observed, regardless of radiolabelling method, makes this possibility improbable in many cases) or from EV components that are attached to the radiotracer. Interestingly, the supplementary data reported by Banerjee *et al.* 90, showing that EV purification by combining ultracentrifugation with SEC leads to lower accumulation in urine, seems to support this hypothesis **(Fig. 11B)**. Previous studies have shown that sEVs isolated by ultracentrifugation can lead to co-precipitation of serum proteins (such as albumin) 120, with lower purity as determined by the particle-to-protein ratio, compared to SEC 121 despite co-isolation of lipoproteins using this method 122. Wei *et al.* have shown that by combining these two methods, it is possible to improve elimination of contaminating proteins, lipoproteins and to improve particle-to-protein ratio 123. Nevertheless, further evidence is needed to determine if this is the case for kidney/bladder/urine uptake of EVs.

Another of the main issues with many EV radiolabelling studies reviewed above is the use of instant thin layer chromatography (iTLC) to measure radiochemical stability in serum. When using appropriate stationary and mobile phases, for example Whatman N^o^ 1 paper as the stationary phase and ethyl acetate or EDTA as the mobile phase for [^89^Zr]Zr-oxinate, the lipophilic unbound radiotracer or leaked ^89^Zr^4+^ ions migrates to the solvent front (R_f_ = 1) respectively, whereas radioactivity bound to the EVs stays at the origin (R_f_ = 0) because EVs are not soluble and precipitate in this solvent system. This technique is appropriate when assessing radiochemical stability in aqueous buffers such as PBS. On the other hand, in serum, any trans-chelation of the radiotracer by the serum proteins would also be detected at the origin because proteins will also precipitate in the presence of organic solvents, even at low concentrations; making it difficult to distinguish whether the radioactivity is bound to EVs or serum proteins.

## Conclusions and perspectives

The field of EV research and in particular the applications of EVs as nanomedicinal tools has sparked a recent remarkable interest from many researchers. From all the research carried out to this effect, it is clear that using imaging techniques, whether optical or radionuclide-based, is certainly beneficial in elucidating EV biology and behaviour, and should be integrated into this research early in the process to facilitate their development as potential therapies/diagnostic tools.

In this review we focussed on EV research to date that includes radionuclide-based imaging, and in particular on the radiolabelling methods used and findings from SPECT/PET imaging. In these studies, two main categories of radiolabelling methods were used - intraluminal and membrane-based. Our review illustrates the importance of choosing the most appropriate radiolabelling method suitable for the downstream application, as each have their advantages and disadvantages. Of all these radiolabelling methods, two main ones stand out in terms of efficiency and stability: *(i)* direct conjugation of a bifunctional radionuclide-complex (*e.g.* NOTA-maleimide) onto surface components and *(ii)* the use of ionophores such as oxine for intraluminal labelling. Ideally, studies that compare these two methods such as that from Faruqu *et al.* should be performed prior to any PET/SPECT EV study to identify the best suited method for each EV and application 84.

It is important to emphasise that when radiolabelling EVs *via* the surface/membrane, we will be tracking these membrane components over time, whether they are still a part of the EV or not. Likewise, when we radiolabel intraluminally we will only be tracking the EVs as long as their contents have not been released. For this reason, regardless of the radiolabelling method, it is important to have a clear understanding of the biodistribution/pharmacokinetics of the radionuclide and/or the radio-complex used for EV radiolabelling. We think this is a crucial component of any EV PET/SPECT imaging experiment as many radionuclides/radio-complexes share biodistribution and excretion pathways with nanoparticulates (*e.g.* liver, lymph nodes, tumours) 69. Ideally, the radiolabelling method of choice should not affect the natural properties of EVs (*e.g.* hydrodynamic diameter, membrane protein structure and composition, colloidal stability), and result in physicochemically and radiochemically stable products. In this regard, we believe there is a significant risk when using direct attachment of radionuclides or radionuclide-based bifunctional chelators onto the surface of EVs, as these are likely to affect the structure of important surface proteins/molecules that the EVs use for their function.

One of the main findings of this review is the high number of studies that show accumulation of imaging signal/EVs in the bladder, making it unlikely that this is simply due to impurities from the radiolabelling process. As we discussed in the previous section, we believe that this may be linked to small EV fragments as a result of fast EV decomposition. Further studies are required to test this hypothesis, and we believe that radionuclide imaging will play an important role in evaluating this.

Finally, it is commonly accepted in the EV field that even a carefully isolated pure microvesicle or exosome population can be highly heterogeneous, and contains subgroups of vesicles with distinct properties 26, 124, 125. This highlights the benefits of integrating *in vivo* EV research with radionuclide-based imaging (that benefits from high sensitivity and quantification properties) to allow us improving our understanding of the basic EV biology of each of these subgroups and their interaction with other cells/tissues. In addition, being a clinically available imaging modality offers the possibility to develop/optimise the methodology for potential future clinical studies.

## Figures and Tables

**Table 1:** Differences in clinical SPECT and PET imaging. Data in the table is collated from Rahmim *et al.* 65 and James *et al.* 56.

	Detection method	Geometric efficiency (percentage of detected to emitted γ rays)	Temporal resolution	Spatial resolution	Sensitivity (concentration of radiotracer needed)
**SPECT**	Collimator detection	~ 0.01%	Minutes	8-10 mm	10^-10^ to 10^-11^ M
**PET**	Coincidence detection	~ 1%	Seconds - minutes	5-7 mm	10^-11^ to 10^-12^ M

**Table 2:** Summary of reports of EV radiolabelling with SPECT radioisotopes. The hydrodynamic size of EVs are stated for unmodified EVs before radiolabelling, as appropriate. Radiolabelling condition column shows EV and radiotracer incubation time, temperature, and the amount of EVs used per reaction. Data shown as reported by the authors. RLY = radiolabelling yield, UC = ultracentrifugation, UF = ultrafiltration, SEC = size exclusion chromatography, RT = room temperature, iTLC = instant thin layer chromatography; * data taken from the figures.

Type	Radionuclide	Source of sEVs / EMVs; size; isolation method	Radiolabelling condition	Purification	RLY	*In vitro* stability; assessed by	*In vivo* imaging	Ref.
Surface radiolabelling	**^125^I**-biotin	B16-BL6 mouse melanoma cells; 70 ± 3 nm; UC	30 min; 37°C; 10 µg	Not reported	~ 80%*	> 95% serum stability at 4 h; UF	û	78
			As above				û	79
	Na**^131^I** + iodo-bead method	Various	30 min; RT; not reported	UF = Nanosep 100k Omega	>80% for 4T1 EVs*	*ca.* 80% serum stability at 24 h*; iTLC	√	80
	**^99m^Tc**-tricarbonyl	Human red blood cells; 188 ± 11 nm; UC + UF + SEC	30 min; RT; 0.6 mL	Zeba spin desalting column	38.8 ± 6.2%	No data given	√	81
	**^99m^Tc**-tricarbonyl	HEK 293T human embryonic kidney cells; ~77 nm; not reported	1 h; 37°C; 20 µg	None used	> 98%	96% saline stability at 24 h; TLC	√	82
	**^99m^Tc** (+ SnCl_2_)	Goat milk; 122 ± 1 nm; UC and SEC	30 min; 37°C; 75 µg	Exosome spin column	37 ± 9%	95% PBS stability at 48 h; iTLC	√	83
	**^111^In**-DTPA	B16-F10 mouse melanoma cells; 132 ± 6 nm; UC	30 min; 37°C; 1x10^11^ sEVs	SEC = Sepharose CL-2B	19.2 ± 4.5%	86.8 ± 3.1% PBS stability, and 80.4 ± 1.6% serum stability at 24 h; iTLC	√	84
Intraluminal radiolabelling	**^111^In**-oxinate	PC3 human prostate cancer cells; 140 ± 59 nm; UC	20 min; RT; 2.5-3.7 mg/mL	SEC = P6 column	81%	No data given	û	85
		MCF-7 human breast cancer cells; 130 ± 57 nm; UC			67%			
	**^111^In**-oxinate	HEK 293 human embryonic kidney cells; 106 ± 14 nm; UF + UC	30 min; RT; 1x10^9^ sEVs/mL	UF = Amicon ultra 100 kDa	> 98%	> 92% serum stability at 24 h; iTLC	√	86
	**^111^In**-tropolone	B16-F10 mouse melanoma cells; 132 ± 6 nm; UC	20 min; 37°C; 1x10^11^ sEVs	SEC = Sepharose CL-2B	4.7 ± 0.4%	43.4 ± 10.1% PBS stability, and 14.2 ± 2.8% serum stability at 24 h; SEC	√	84
	**^99m^Tc**-HMPAO	Raw 264.7 mouse macrophages; 218 ± 8 nm; serial extrusion	1 h; RT; 100 µg	SEC = PD-10, MW3000 spin column	> 93%	~ 90% serum stability at 5 h; iTLC	√	87
	**^99m^Tc** (+ SnCl_2_)	Rat red blood cells; 201 ± 16 nm; serial extrusion	20 min; 37°C; 100 µg	UC (only if RLY < 95% on iTLC)	100%	93 ± 3% serum stability at 24 h; iTLC	√	88

**Table 3:** Summary of reports of EV radiolabelling with PET radioisotopes. The hydrodynamic size of EVs are stated for unmodified EVs before radiolabelling, as appropriate. Radiolabelling conditions column shows EV and radiotracer co-incubation time, temperature, and the amount of EVs used per reaction. Data shown as reported by the authors. RLY = radiolabelling yield, UC = ultracentrifugation, SEC = size exclusion chromatography, RT = room temperature, iTLC = instant thin layer chromatography; * data taken from the figures.

Type	Radionuclide	Source of sEVs; size; isolation method	Radiolabelling conditions	Purification	RLY	*In vitro* stability; assessed by	*In vivo* imaging	Ref.
Surface radiolabelling	Na**^124^I** + iodogen method	MLP29 mouse liver cells; ~130 nm; UC	2 h; 25°C; 0.4 µg	SEC = Sephadex G25 (DNA grade)	Glycosylated = 17 ± 2% Non-glycosylated = 19 ± 1%	> 90% PBS stability at 72 h; iTLC	√	89
	**^64^Cu**-DOTA	Human umbilical cord blood mononuclear cells; ~110 nm; UC	1 h; RT; > 300 µg	MW3000 exosome spin column	16 - 25%	94% serum stability at 24 h, 95% blood stability at 1 h; iTLC	√	90
	**^64^Cu**-NOTA	4T1 mouse breast cancer cells; 106.3 ± 0.3 nm (volume weighted); UC	30 min; 37°C; 300 µg	SEC = PD-10	Non-PEGylated = 91.2 ± 0.2% PEGylated = 85.7 ± 0.7%	Non-PEGylated = 80.4 ± 1.3% PEGylated = 95.7 ± 0.9% serum stability at 24 h; iTLC	√	91
	**^64^Cu**-NOTA-Cy7	4T1 mouse breast cancer cells; ~100 nm; ExoQuick^®^	5 min; 37°C; 100 µg	ExoQuick^®^	~ 98%	> 95% serum stability at 36 h*; iTLC	√	92
	**^68^Ga**-NOTA-Cy7		30 min; 25°C; 100 µg		Not reported	Not reported		
Intraluminal radiolabelling	**^89^Zr**-oxinate	B16-F10.GFP mouse melanoma cells; 146 ± 2 nm; ExoQuick^®^	1 h; 37°C; 1x10^10^ sEVs	MW3000 exosome spin column	6 ± 1%	Not reported	û	93
		MDA-MB-231.CD63-GFP human breast cancer cells; 121 ± 14 nm; UC	20 min; 37°C; 1x10^10^ sEVs	SEC = Sepharose CL-2B	6 ± 1%		û	
		PANC1 human pancreatic cancer cells; 97 ± 4 nm; UC	20 min; 37°C; 1x10^11^ sEVs		23 ± 7%	76 ± 3% PBS stability at 26 h; iTLC (37°C)	√	
