# Supplementary material for: Radiolabelling of Extracellular Vesicles for PET and SPECT imaging
Source: Nanotheranostics. 2021 Feb 13;5(3):256–74. doi: 10.7150/ntno.51676 (PMC7914338; doi:10.7150/ntno.51676)
Supplement: Supplementary file 1 — Supplementary materials. [file ntnov05p0256s1.pdf]

Supplementary Information for:

Radiolabelling Extracellular Vesicles for PET and SPECT imaging

Azalea A. Khan and Rafael T. M. de Rosales*

Dept. of Imaging Chemistry and Biology
School of Biomedical Engineering and Imaging Sciences
King's College London
St. Thomas' Hospital,
London, United Kingdom

*E-mail: rafael.torres@kcl.ac.uk

Methodology

In order to identify articles on EV radiolabelling to date (July, 2020), search was performed on 'Web of Science' database using 'exosom*', 'microvesic*', 'EVs', 'extracellular vesicle*' as topics in combination with 'positron emission tomography', 'PET', 'single photon emission computed tomography', 'SPECT', 'radiolabel*', 'radionuclide imaging' and 'nuclear imaging'. Conference abstracts, reviews and book chapters were excluded. Separate searches were performed on the pre-print servers, chemRxiv and bioRxiv, using the same terminologies as above. A total of 16 published articles were found and analysed in this review. Of these, 2 articles were found on bioRxiv and chemRxiv each. 11 of those articles were on SPECT imaging and 5 of them were on PET imaging, and was analysed to create Figure 3.
